# Genome-Wide Gene-Set Analysis Approaches in Amyotrophic Lateral Sclerosis

**DOI:** 10.3390/jpm12111932

**Published:** 2022-11-20

**Authors:** Christina Vasilopoulou, Stephanie Duguez, William Duddy

**Affiliations:** Personalised Medicine Centre, School of Medicine, Ulster University, Londonderry BT47 6SB, UK

**Keywords:** amyotrophic lateral sclerosis, genome-wide association studies, ALS pathology, gene-set analysis, functional genomics

## Abstract

The rapid increase in the number of genetic variants identified to be associated with Amyotrophic Lateral Sclerosis (ALS) through genome-wide association studies (GWAS) has created an emerging need to understand the functional pathways that are implicated in the pathology of ALS. Gene-set analysis (GSA) is a powerful method that can provide insight into the associated biological pathways, determining the joint effect of multiple genetic markers. The main contribution of this review is the collection of ALS GSA studies that employ GWAS or individual-based genotype data, investigating their methodology and results related to ALS-associated molecular pathways. Furthermore, the limitations in standard single-gene analyses are summarized, highlighting the power of gene-set analysis, and a brief overview of the statistical properties of gene-set analysis and related concepts is provided. The main aims of this review are to investigate the reproducibility of the collected studies and identify their strengths and limitations, in order to enhance the experimental design and therefore the quality of the results of future studies, deepening our understanding of this devastating disease.

## 1. Introduction

Amyotrophic Lateral Sclerosis (ALS) is a rare, motor neuron disease that is primarily characterised by the loss of upper and lower motor neurons. The peak age of onset of the disease is at 54–67 years old, although onset may occur at any age [1,2,3,4]. ALS is progressively fatal, with typical survival of 2–5 years after the onset of the first symptoms; however, 5–10% of the affected individuals survive more than 10 years [1,5,6]. Not only do we lack a mechanistic understanding of ALS, but its prevalence is increasing with the ageing of the world population [2,7], thus there is an increasing need to understand ALS pathology and the underlying molecular pathways.

In recent years, discoveries of multiple Genome-Wide Association Studies (GWAS) to ALS have provided new insights into the disease susceptibility and pathology [8,9,10]. As of September 2022, the GWAS Catalog has published 345 variants and risk allele associations with ALS [11]. Variants located in more than 30 genes have been discovered to be associated with a high risk of ALS [12,13,14,15,16]. The first ALS-associated mutations were discovered in the Cu/Zn superoxide dismutase 1 gene, *SOD1*, explaining 20% and 2% of familial and sporadic ALS, respectively [17]. More recently, the hexanucleotide GGGGCC (G4C2) repeat expansion (HRE) located within the first intron of the *C9orf72* gene was characterised as the most frequent cause of both familial and sporadic ALS [18]. Other genes linked to ALS include fused in sarcoma *FUS*, and transactive response DNA-binding protein of 43 kD *TARDBP/TDP-43* [12]. Thus far, evidence supports a model implicating rare variants along with non-genetic causes, such as environmental factors [4,19,20,21]. However, large GWAS studies have suggested an intermediate genetic architecture for ALS that falls somewhere in the middle of the spectrum of genetic pathology in terms of effect size and prevalence of risk variants lying between conditions such as schizophrenia (many common variants each small effect sizes), and Huntington’s disease (rare large-effect variants located in a single gene) [4,22,23,24,25].

Despite the fact that GWAS has identified multiple risk variants and ALS-associated genes, our knowledge is very limited in terms of the affected functional ALS pathways and the underlying pathology. Recent systematic reviews have aimed to explain ALS pathology, through the investigation of the molecular pathways that are implicated in ALS based on the collective knowledge and functional interpretation of multiple known ALS-associated genes [12,25,26,27]. However, the discovery and analysis of the functional processes that are implicated in ALS, through the review of the known ALS-associated genes is a complex task, burdened by the heterogeneity of the disease [12,26].

Genome-wide gene-set analysis (GSA), also known as enrichment and pathway analysis, is an emerging powerful strategy to understand the genetic contribution to the phenotype in terms of the impact of genetic variants on biological pathways, using GWAS summary statistics or individual-based genotype data [28,29]. In GSA, individual Single Nucleotide Polymorphisms (SNPs) are summarized to whole genes, taking into account the associations of multiple genetic markers, and genes are then summarized into gene sets [29]. A gene set is any group of genes that share a common attribute. This attribute can be, among others, a biological pathway, a network module, or a group of interacting components, depending on the biological hypothesis. The aim of gene-set analysis is to test the association between a gene set and a particular phenotype.

The scope of this review covers genome-wide association studies that employ gene-set analysis in order to uncover biological mechanisms that are statistically associated with ALS. The databases of PubMed and Google Scholar were used to identify relevant peer-reviewed papers using the terms “amyotrophic lateral sclerosis”, “GWAS” and “gene-set analysis”. The review is structured as follows: first, a summary of the limitations in single-gene analysis is presented; then, a brief overview of the main statistical properties and characteristics of GSA is provided, leading into a description and comparison of the published gene-set analysis studies using ALS GWAS datasets to-date.

## 2. Limitations on Single-Gene Analysis

GWAS is a single marker analysis, testing independently the association of each single variant to a phenotype. The first step towards the functional interpretation of a GWAS study is to map genomic variants to genes, usually adding a window of 10–25 kb upstream and downstream of each gene to also include associated regulatory regions of the gene. After the gene mapping, tens of thousands of single-gene tests are performed to define their contribution to the phenotype. Then, a portion of “significant” genes is chosen for further interpretation and analysis. Single-gene analysis studies present a number of limitations. Such limitations have been outlined in several review papers aiming to outline challenges, approaches and future advances in gene expression gene-set analysis [28,30]. Genome-wide GSA has derived from gene expression gene-set analysis, and it has been shown that the two types of analysis have the same statistical properties [29]. We interpret some of these limitations below under the prism of GWAS and incorporate key arguments collected from related papers.

One common limitation present in both GWAS and single-gene/gene-level analyses is the need for multiple testing correction strategies. GWAS and gene-level analyses are both univariate analyses, testing millions or tens of thousands of associations of single variants or genes, respectively, one at a time, under the assumption that each association test is an independent event. In GWAS studies, it is standard practice to correct for family-wise type I errors, using the Bonferroni correction. In gene-level studies, both conservative and lenient methods, like Bonferroni and False Discovery Rate (FDR), are followed to correct for multiple testing errors. However, being too conservative or too lenient can lead to the exclusion of false negatives and the inclusion of false positives in the final table of results, respectively. The choice of which threshold to apply can differ among authors, and this can contribute to low reproducibility results among studies, as different thresholds are expected to lead to a different final results table of significant genes, and thus to different interpretations [28].

Another challenge in single-gene analysis relies on the interpretation as well as a potential bias on the final limited table of potentially hundreds of “risk”/“significantly- associated” genes, depending on the threshold or the selection process. An obvious problem in this case is the *curse of dimensionality* where the researcher needs to interpret and analyse hundreds to thousands of “interesting” features (i.e., genes). Another challenge arises from the fact that it is common for a certain amount of genes to be involved in multiple and different biological pathways. These genes are called multi-functional and are estimated to make up for the 26% of the overall annotated genes in *Homo sapiens* [28,31]. Thus, the gene interpretation increases in complexity, and it is vulnerable to introducing a hypothesis-driven bias which can lead to false conclusions [28].

Single-gene analysis cannot reveal functional groupings of multiple disease-associated genes [28]. Various types and sources of functional groupings of genes exist, including among others, biological pathways, cellular components, and disease phenotypes, derived from biological annotation databases such as the Molecular Signatures Database (MSigDB) [32,33], functional gene sets from the Gene Ontology (GO) [34], Kyoto Encyclopedia of Genes and Genomes (KEGG) [35] and known disease-gene lists from DisGeNET [36,37].

## 3. A Brief Overview of Gene-Set Analysis

Genome-wide GSA can overcome the previous limitations. Firstly, in a genome-wide GSA, significance thresholds or hypothesis-derived exclusion criteria are not necessary in the SNP-level or the gene-level analysis, in order to reduce the feature space. However, in the following sub-Chapters and in the Discussion section, we mention ALS GSA studies and GSA software that follow this approach at the gene level in order to narrow the number of GSA results. In addition, the interpretation of the results becomes more straightforward when the analysis is focused on several functional pathways. Lastly, we know that real-life biological systems are multi-layered complex networks. Multiple genes are involved in biological processes. GSA allows multiple subtle gene associations to emerge in a synergistic way through a grouping of genes that share common attributes. Such subtle/nominal gene associations would probably be discarded or overlooked as false negatives in a single-gene analysis.

Previous review papers have been published with the aim to classify and evaluate gene-set analysis methods as well as to categorise and elaborate on the different null hypotheses and properties of each model. An excellent review that focuses on the statistical properties and structure of genome-wide GSA is by De Leeuw et al. [29]. In this sub-chapter, the main structure of GSA is summarized, and the main properties that categorise GSA methods are mentioned; in addition, confounding factors that can affect the results of GSA are discussed. The elements above are used to group and compare the GSA methods employed by the collected ALS genome-wide GSA studies.

### 3.1. The Structure of Gene-Set Analysis

The aim of GSA is to calculate the association between a gene set and the phenotype of interest. Here, we define a gene set as any group of genes that share a common attribute; some examples include groups of genes that participate in the same biological pathway, cellular component, or are linked to a specific disease or phenotype. This a priori knowledge can be mined through biological databases such as MSigDB [32,33] or even can be predicted using computational approaches like Machine Learning [25].

There is an extensive catalogue of published GSA/enrichment analysis software; however, the core structure behind these tools is the same. GSA is divided into two main stages:

- Each SNP is assigned to a gene—using specific annotation files in order to map each SNP into a gene region based on a kilobase window around the gene so that the researcher can additionally include regulatory elements—and each gene is then tested for its association with the phenotype.
- Genes are mapped to gene sets, and an association measure is computed for each gene set.

After the first step, a gene-level matrix is constructed where the unit of analysis is the genes. Each row in this matrix is a gene association measure and a label feature representing whether gene X is part of gene set A (1: Gene X belongs in gene set A, 0: Gene X does not belong in gene set A) [29]. When this matrix is complete, GSA conducts a bivariate test between the label feature and the gene-level association measures [29].

### 3.2. Main Categories of Gene-Set Analysis Methods

The core GSA structure, different null hypotheses, gene/gene-set statistical measures, and confounding factor correction strategies, among others, can all vary between GSA approaches. This leads to differences in accuracy and power, and also in the biological interpretation of results.

There are two main null hypotheses that differentiate GSA methods, which both determine the statistical test on the gene-level matrix and affect the interpretation of the results [38]. The first is called *competitive*. The competitive hypothesis considers all the genes and tests whether the joint association of genes within a gene set A is greater than the association of genes that do not belong in this gene set. The second null hypothesis is called *self-contained*—it considers only the genes within a gene set A and tests if the joint gene association has any effect on the phenotype at all [29,38]. The choice of the null hypothesis affects the interpretation of the results fundamentally. Self-contained GSA provides information only about the genes within the gene set, whereas competitive GSA acknowledges the association signal of all genes and tests a hypothesis that can result in biological meaningful conclusions [29]. However, competitive GSA is vulnerable to a number of confounding factors like linkage disequilibrium (LD) [29]. De Leeuw et al. [29] compared and evaluated a number of GSA methods and showed that competitive models implemented in MAGMA (Multi-marker Analysis of GenoMic Annotation) and INRICH (INterval enRICHment analysis) show good statistical performance, accounting for a number of confounding factors.

A second attribute relates to the gene test statistic representation/computation among GSA software. The selection of gene association measure relates to assumptions of the underlying genetic architecture of the phenotype of interest [29]. The gene association is represented by *p*-values or transformed *p*-values, usually computed through functions using the mapped SNP *p*-values. A common approach has been to assign the highest associated SNP *p*-value to represent the mapped gene association [29]. Some other approaches include computing the mean association of all the mapped SNPs, or the mean association of only the “top” SNPs that are mapped within a gene. MAGMA, a widely used gene-set analysis command-line tool [39], integrates alternative gene association measures including the SNP-wise multi-model where each gene is represented by the weighted mean/sum of multiple models (e.g., mean SNP association, top SNP association) as well as Principal Components Regression (PCR) where the disease phenotype is regressed on the principal components of all mapped SNPs in a gene [39]. However, the only accepted input of PCR is individual-level genotype data in a binary format used by PLINK software, so imputed dosages/probabilities can not be used in this model yet. The SNP-wise multi-model can be an ideal choice when the underlying genetic architecture of the phenotype is not known, since it combines multiple models with different strengths and sensitivities.

A third important GSA characteristic that differentiates GSA methods, in terms of statistical power, is the gene-set test statistic. The gene-set test statistics are categorised in the following classes:

- *Mean-based*, where the gene-set association measures are summarized using the mean or sum of the gene associations.
- *Count-based*, where the genes are labelled as “significant” or “not significant”, and only “significant” genes determined by a specific cut-off are considered in the gene-set test statistic.
- *Rank-based*, where the genes in the gene-level matrix are ranked by their association with the phenotype and then an overrepresentation of the genes that belong in the gene set and also are at the top of that ranking is computed.

De Leeuw et al. [29] compared these methods through various simulations and showed that mean-based methods demonstrate more powerful results than the rank- and count-based methods. A reason for this loss of power in rank- and count-based methods is due to a loss of information after the ranking and the categorisation of “significant” and “not significant” gene sets based on a chosen cut-off [29].

### 3.3. Gene-Set Analysis Confounding

A number of confounding factors can affect the statistical performance of GSA methods and lead to biased and false-positive results. Linkage disequilibrium (LD) is one factor that has an effect on the SNP and gene-level association. SNPs, and therefore genes that are inherited together because they are localised in the same genomic region, are in LD and are therefore correlated. These gene–gene correlations need to be accounted for, so as to discern a true gene association to the phenotype from association signals that stem from genes that are in high LD with this true-causal gene [28,29]. The amount of LD among mapped SNPs within a gene is called gene density. Another common confounding factor is gene size, representing the number of SNPs that are mapped within a gene. The latter becomes evident when a gene is chosen to be represented by the highest associated mapped SNP; larger genes that contain more SNPs have a higher probability to contain a more highly associated SNP by chance, in comparison with smaller genes [29].

GSA methods are also prone to population stratification [29], as each population with a different ancestry is expected to have different allele frequencies, so using a heterogeneous input GWAS cohort can affect the detection of true gene-set associations to the phenotype. In this context, it is also important that accurate and comprehensive quality control strategies are followed in the input genomic dataset, prior to the GSA, so that only high-quality samples and variants are used for subsequent analysis [40].

## 4. Recent Approaches in ALS Genome-Wide Gene-Set Analysis Studies

This section aims to summarize, analyse and compare the experimental design and the results of ALS GWAS studies that employ gene-set analysis as a method for the discovery of functional biological pathways that have a statistically significant association with ALS. Although some of the collected studies aim to understand the pathology of ALS using a variety of genomic approaches and address multiple research questions, the main focus of this section is on gene-set analysis results and methodologies. The main inclusion criteria for the collection of the studies were to use ALS genomic data (either GWAS summary statistics or individual-level genotype data) and to employ gene-set analysis for the discovery of statistically associated groups of genes to ALS. For the latter reason, ALS studies that used gene-set analysis tools only for gene-level results were not considered. The structure of this section is the following: first, the collected studies are categorised based on common features of their experimental design, and then the results of each study are summarised. The ALS gene-set analysis results are compared while investigating their reproducibility in the Discussion section.

In total, nine studies were considered relevant, shown in Table 1. The studies were categorised based on their input data, meaning GWAS summary statistics or individual-level genomic datasets and/or other types of data like expression Quantitative Trait Loci (eQTLs) that were used as input for the gene-set analysis software. Specific features like the number of ALS cases and controls, adding the citation of each specific study that published the input data for further information, as well as the ancestry of the input data, are mentioned. Furthermore, the studies were compared in terms of the employed gene-set analysis software and their different algorithms (described in more detail in Table 2). Lastly, the source/database of the collected gene sets is included in the last column of Table 1.

Van Rheenen et al. [41] conducted the largest ALS cross-ethnic GWAS combining European, Japanese and Chinese ancestry genomic data. The study conducts disease-relevant cell and tissue-specific enrichment analysis tests on European ancestry summary statistics, using FUMA [54]. FUMA incorporates MAGMA for the gene-level *p*-value calculation and then tests if the expression of these genes is particularly enriched for tissues and cell types using gene expression patterns from the Genotype–Tissue Expression (GTEx) [41,54]. The cell type-specific enrichment analyses included single-cell RNA-seq datasets of human-derived brain samples using FUMA and showed statistically significant enrichment for neurons [41]. False Discovery Rate (FDR) was used as a multiple testing correction strategy in the tissue and cell type enrichment gene-set analyses with a threshold of FDR < 0.05 [41]. In addition, the authors followed an extra gene-set analysis approach using Downstreamer incorporating gene-level associations with multi-tissue and brain-specific gene co-expression matrices [41,55]. The authors report ALS-related statistically significant gene sets from the Human Phenotype Ontology (HPO) using the brain-specific gene co-expression matrix, passing the Bonferroni multiple testing correction, including cerebral cortical atrophy, abnormal nervous system electrophysiology and distal amyotrophy [41]. Lastly, the authors tested for statistically significant biological processes using Reactome and Gene Ontology gene sets. After Bonferroni multiple testing correction, the brain-specific coexpression enrichment analysis identified membrane trafficking, intra-Golgi and retrograde Golgi-to-endoplasmic reticulum (ER) trafficking and macroautophagy as statistically significant gene sets that are associated with ALS [41].

Benyamin et al. [42] conducted a cross-ancestry meta-analysis to a large European ancestry summary statistics dataset [20], and to in-house generated Chinese ancestry genomic data. They also used two Australian descent replication cohorts to validate their results. The combined cohort revealed a novel ALS-associated locus, spanning the genes glutathione peroxidase 3 (*GPX3*) and tumor necrosis factor alpha-induced protein 3-interacting protein 1 (*TNIP1*), a finding that was also replicated in the two Australian cohorts [42]. GPX3 is an antioxidant molecule and shares a functional link with the superoxide dismutase 1 (*SOD1*), a gene whose various mutations have been previously associated with ALS [56,57]. *TNIP1* is also known to interact with the known ALS-associated gene optineurin (*OPTN*) [56], and has also been associated with inflammation [42]. However, the study did not identify any statistically significant biological pathway that was associated with ALS.

Saez-Atienzar et al. [45] followed a polygenic risk score (PRS) approach to gene-set analysis using PRSice-2 [58]. Polygenic risk scores combine multiple variants to calculate a part of an individual’s susceptibility to a particular phenotype, interpreted as the weighted sum of the number of risk alleles for each individual [59]. Briefly, the authors used a reference summary statistics dataset [20] to define the weights of risk alleles; then, these risk allele weights were used on a second training individual-based genomic dataset [14], in order to calculate PRS estimates on biological gene sets [45]. Lastly, they used a third testing set of individual-based genotype data to validate their results [45]. The authors used three gene-set categories from MSigDB, including the hallmark, curated and Gene Ontology gene sets [45]. Out of the 7296 MSigDB gene sets, the authors report 13 statistically significant gene sets that were replicated across their training and testing genomic sets [45]. These 13 pathways after semantic similarity analysis are represented by the following biological categories: neuron projection morphogenesis, membrane trafficking, and signal transduction mediated by ribonucleotides [45].

Several of the other collected studies combined large ALS GWAS data with expression Quantitative Trait Loci (eQTL) data, using Summary data-based Mendelian Randomization (SMR). SMR integrates GWAS and molecular traits data like gene expression, to test the chance that SNPs that increase the risk of a disease do so through modifying gene expression [44,60]. Iacoangeli et al. [44] employ a large GWAS meta-analysis dataset as well as publicly available eQTLs for the frontal cortex, cortex, cerebellum and cerebellar hemisphere. The study identifies *SCFD1* as the only statistically significant gene that seems to increase ALS risk though eQTLs (SMR *p*-value = 4.29 × 10^−6^) [44]. SCFD1 is a Section 1/Munc18 (SM)-like protein localized in the autolysosome that plays a key role in SNARE complex formation and autophagosome-lysosome fusion [61], as well as in retrograde Golgi-to-endoplasmic reticulum (ER) transport [62]—processes that have been previously linked to ALS pathology. The functional enrichment analyses were conducted on a subset of 382 genes, which were deemed to show levels of association with the *SCFD1* trans-eQTL hotspot [44]. For their gene functional enrichment analyses, they used methods like the Enrichr, gProfiler, and GSEA [63,64,65]. The authors identified various processes such as the retrograde vesicle-mediated protein transport from the ER-to-Golgi, glutamatergic synapse and the regulation of synaptic vesicle docking and exocytosis, to be statistically relevant to *SCFD1* eQTL expression [44]. Du et al. [46] also use SMR to combine ALS GWAS meta-analysis data [20] with eQTLs [47]. The authors subjected the SMR results to pathway analysis using the proposed GSEA method of Wang et al. [65] and 162 biological pathways from the KEGG database [46]. They report seven ALS-associated KEGG pathways, including peroxisome, citrate cycle (TCA cycle, Krebs cycle), tight junction, PPAR signaling pathway, SNARE interactions in vesicular transport, arachidonic acid metabolism, and glycolysis-gluconeogenesis [46]. The study conducted 5000 permutations to calculate empirical *p*-values, although details are not provided concerning any multiple testing correction of the gene-set analysis results.

In Table 1, two early GSA studies are listed that employ the same input GWAS datasets, having a small cohort size of American and Irish descent. The most recent study is by Deng et al. [48], who conducted an ALS multi-ancestry functional enrichment study to identify reproducible ALS-related genetic factors. The authors use the American and Irish GWAS summary statistics datasets to map SNPs to genes using a 20 kb window upstream and downstream of each gene [48]. Each gene was represented by the minimum mapped SNP *p*-value. Then, they filtered the mapped genes in each dataset using a *p*-value < 0.01 threshold and subjected those genes to gene-set analysis using WebGestalt (WEB-based GEne SeT AnaLysis Toolkit) [66], an overrepresentation software performing hypergeometric tests [48]. They report 34 Gene Ontology biological processes shared from the Irish and the American studies [48]. Lastly, the authors report the nervous system developmental pathway as the most associated with ALS pathology, as it was related to the majority of the identified ALS-associated pathways [48]. Furthermore, Shang et al. [52] employed the same American and Irish descent datasets to conduct enrichment analysis using also WebGestalt for overrepresentation testing. That study used the ProxyGeneLD to calculate gene-level associations, taking into account linkage disequilibrium (LD) patterns as well as correcting for other confounding factors such as gene length [67]. The authors further filtered their gene pool by applying a *p*-value < 0.05, leaving 1124 and 897 genes in the American and the Irish datasets, respectively [52]. These genes were used as input for the WebGestalt software as well as KEGG pathways for gene set annotation. The authors used FDR for a multiple testing correction (FDR < 0.05), and they removed pathways that contained less than 20 genes and more than 300 genes in order to avoid testing overly narrow or broad gene sets [52]. The authors report 50 and 45 statistically significant pathways in the American and Irish cohorts, accordingly [52]. The 12 shared significant pathways were related to metabolism, immune system and diseases, environmental information processing, genetic information processing, cellular processes, nervous system and neurodegenerative diseases [52].

In addition, Lee et al. [51] employ the same American dataset [49] as Deng et al. [48] and Shang et al. [52], to identify SNPs, genes and pathways that have a statistically significant association with ALS through the ICSNPathway web server [51,68]. First, the ICSNPathway uses iGSEA (improved Gene-Set Enrichment Analysis), a rank-based GSA algorithm which is conducted on the overall GWAS *p*-values where SNPs are mapped to genes, and each gene is represented by the lowest SNP *p*-value. Then, the genes are ranked by their *p*-value and the algorithm measures the tendency of the genes of a pathway to be located at the top of the ranked gene list [68]. The study uses KEGG, BioCarta and GO molecular function and biological processes for functional annotation of the gene sets, and applied a minimum of 5 and a maximum of 100 genes threshold for each gene set [51]. They conducted a limited QC analysis on the individual-level genomic dataset applying thresholds of Hardy–Weinberg equilibrium and variant call rate [51]. The highest significantly associated gene sets were chromatin and nucleosome assembly [51].

Lastly, Xie et al. [53] conducted a pathway analysis to a Chinese Han descent genomic dataset. The authors mapped 859,311 SNPs to genes and applied a cut-off of *p*-value < 10^−4^, leading to 495 candidate genes [53]. These genes were used as input into the WebGestalt software [66], and the KEGG database was used for gene-set annotation [53]. The authors report 10 significantly associated pathways to ALS (FDR < 0.05), including phosphatidylinositol signaling system, pathways in cancer, Wnt signaling pathway, axon guidance, MAPK signaling pathway, neurotrophin signaling pathway, arrhythmogenic right ventricular cardiomyopathy, colorectal cancer, arachidonic acid metabolism, and T-cell receptor signaling pathway [53]. Details were not provided on the quality control of the in-house generated genomic data.

## 5. Discussion

**Figure 1:**
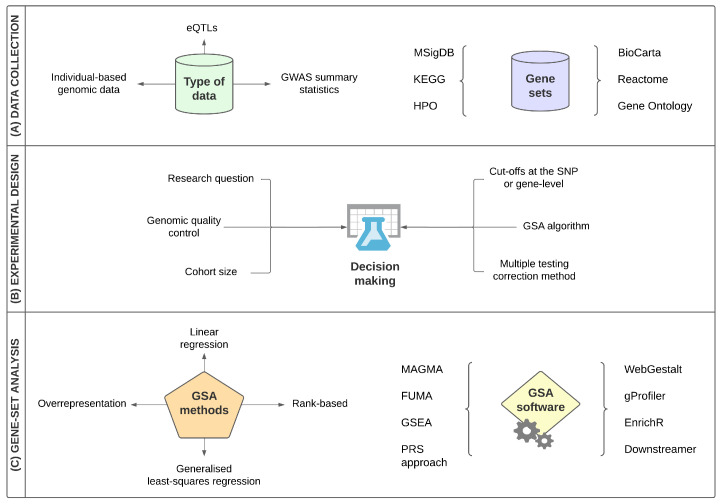
Main strategies and features of the collected ALS gene-set analysis studies. The main GSA design characteristics of the collected studies include (**A**) Collection of the input data that may include individual-based genomic data, summary statistics and any other type of biological data, e.g., expression Quantitative Trait Loci (eQTLs) as well as the collection of gene sets from a variety of annotation databases such as the Gene Ontology, Kyoto Encyclopedia of Genes and Genomes (KEGG) and Molecular Signatures Database (MSigDB); (**B**) decision-making for a variety of steps of the main experimental design, including among others, defining the main research question of the study, following a genomic quality control analysis if individual-based genomic data are analysed, and which gene-set analysis method/algorithm/software and multiple testing correction strategies are more appropriate for the study and (**C**) gene-set analysis main methods and software of the collected studies.

In the present study, we collected nine ALS gene-set analysis studies, in order to compare their methodology and biological results. In this section, we identify the main limitations of the collected studies, compare their experimental design with an emphasis on their gene-set analysis methods and summarise their significant findings, while testing for any potential reproducibility. A brief summary of the collected strategies and gene-set analysis methods is shown in Figure 1.

### 5.1. Cohort Size Affects the Power of Genome-Wide GSA

We note several limitations of the earliest studies published from 2014 to 2017 (listed at the end of Table 1). One limitation derives from the very limited cohort size of the genomic datasets, which inevitably leads to a loss of power in the GWAS results. The most commonly used datasets were of American and Irish descent and consisted of 276 cases/271 controls, and 221 cases/216 controls [49,50], respectively. This is an expected limitation considering that it is only with recent advances in genomic technologies that the costs of sequencing have decreased sufficiently to enable the availability of larger ALS-control genomic cohorts. More recent studies have focused on the two larger GWAS datasets, including the summary statistics of 12,577 ALS cases and 23,475 controls released in 2016 [20], and, in 2018, the public release of the GWAS meta-analysis summary statistics of 10,031,630 imputed SNPs of 20,806 cases and 59,804 controls [14]. In 2021, van Rheenen et al. published the largest cross-ancestry GWAS dataset to date, including 27,205 cases and 110,881 controls of European and Asian descent [41]. We note that, in the context of tissue and cell type enrichment analyses, as well as in biological pathway analyses, van Rheenen et al. used only a European descent GWAS meta-analysis cohort [41].

### 5.2. Limitations on Dimensionality Reduction Approaches

Another limitation, mostly in the earliest studies, concerns dimensionality reduction approaches, prior to the gene-set analysis stage, which aim to reduce the initial number of SNPs/genes to a subset of potential ALS “risk” SNPs/genes, in order to limit the number of gene-set analyses and ease interpretation. The curse of dimensionality is a common challenge in GWAS studies, as modern analytical platforms and imputation strategies lead to datasets containing millions of genetic markers. This challenge not only poses computational problems but also makes it difficult and time-consuming to discern the few variants/genes that are likely to be associated with the phenotype from other putative false-positive results and to further investigate their involvement in downstream events. Furthermore, many genes play multiple roles, participating in several biological pathways [28,31]. Among the previous studies, Deng et al. [48], Shang et al. [52] and Xie et al. [69] apply gene *p*-value cut-offs of 0.01, 0.05 and 0.0001, respectively, to reduce the number of subsequently analysed genes that enter the gene-set analysis stage. Another example of a dimensionality reduction approach from a recent study was followed by Iacongeli et al. [44]. Specifically, the enrichment analyses were limited to a subset of 382 genes which showed a significant level of co-expression with the *SCFD1* gene, the only gene in their analysis that reached statistical significance in increasing the ALS risk through eQTLs [44].

The choice of different thresholds by different authors makes overall conclusions difficult, as this affects the comparability and reproducibility of findings, as different thresholds may lead to different biological results and interpretations [28]. In addition, the filtering of genes prior to gene set analysis risks the exclusion of false-negative genes and gene sets, as well as narrowing the possible scope of conclusions. Lastly, single-gene analysis is not as capable of detecting subtle multi-gene associations in comparison with genome-wide gene-set analysis [28].

### 5.3. Comparing the Collected Gene-Set Analysis Methods

In Table 2, the GSA software tools are summarised based on their type of input data, their null hypothesis, their default gene-set method and the ALS GWAS-GSA studies that used these particular tools. The information about the GSA software tools was retrieved from relevant review papers that categorised several GSA methods [29,30,40], as well as obtained from the original software published papers or tutorial guides (when papers were not available). Table 2 was an effort to summarise the GSA approach of each study, presenting the main features that were employed, rather than exhaustively listing all possible settings that are implemented in each tool. A challenge in this effort was that several studies did not provide details of their GSA approach. In this case, it was assumed that the authors used the default settings of each tool.

We observe that the vast majority of the studies used the competitive null hypothesis and overrepresentation GSA methods. Overrepresentation competitive methods involve the labelling of each gene as “significant” or ”non-significant” based on a specific threshold, and then on the gene-set level, the proportion of “significant” genes within a gene set is compared to the proportion of “significant” genes across the rest of the genes that do not belong in this gene set [40]. The hypergeometric test is a common choice as an association test in the overrepresentation GSA methods. A limitation of overrepresentation methods, which is also common in single-gene analyses as discussed in Section 2, is that there are no gold standard thresholds to determine which genes are significant or not, and these thresholds can influence the GSA results. Another limitation stems from the use of only “significant” genes, as this may lead to a loss of information, and, for this reason, it has been proposed to use GSA methods that employ the whole pool of gene *p*-values for the gene-set analysis [40]. Such examples include MAGMA and GSEA.

A number of studies used the minimum SNP *p*-value of the overall SNPs mapped to a gene to represent a gene, including [44,46,48,51,52,53]. The latter study does not include this information in the paper, but we assume that this is the practice that they followed as this is the default approach in the GSEA algorithm [29,68]. However, recent studies show that employing joint effects of multiple SNPs to model gene effects is more powerful than simply selecting the minimum SNP *p*-value to represent a gene-level statistic [40,70]. Especially when we know very little about the underlying genetic architecture of the disease, a good practice could be to combine multiple gene-level statistic representations. Such an approach is implemented in tools like MAGMA which use a multiple regression model at the gene level and provide the SNP-wise multi-model, which creates an aggregate gene statistic, combining different gene models [39]. It has been previously shown that MAGMA and INRICH provide consistently good performance while accounting for a number of confounding factors, including, among others, gene–gene correlations, as well as gene size and density [29,39]. Two studies used MAGMA, the first of which was Benyamin et al. [42], who unfortunately did not provide detailed information on their GSA experiments, and their approach did not yield any significant results. The second study was by van Rheenen et al. [41] who employed FUMA software that incorporates MAGMA to calculate the gene-level statistics.

It is important to mention that FUMA and Downstreamer tools employ different approaches from standard GSA software and were used to test specific hypotheses by van Rheenen et al. [41]. For instance, FUMA [54] was employed to test for tissue and cell type enrichment using gene expression patterns from the Genotype-Tissue Expression (GTEx) and single-cell RNA-seq datasets [41]. Downstreamer (presently available only in a pre-print format) aggregates SNP association statistics while accounting for LD, and uses multi-tissue or brain-specific coexpression networks to identify disease-associated gene sets that show significantly enriched co-regulation patterns with genes inside the associated GWAS loci [41,55]. Lastly, Saez-Atienzar et al. [45] followed a PRS approach to identify disease-related pathways based on pathway-based polygenic risk score estimates, mapping SNPs directly to gene sets.

### 5.4. Gene-Set Analysis Deepens Our Understanding of the Implicated ALS Functional Pathways

A summary of the main biological pathways discovered by each study is provided in Table 3. We note that the vast majority of the studies used a false discovery rate as a multiple testing correction method to account for family-wise type I errors. However, in some cases, only empirical *p*-values were used for subsequent interpretation and analysis [46]. In this sub-chapter, the significant GSA results of each study are compared and grouped by the main characteristics of each study design.

Several of the collected studies combined European ancestry ALS GWAS summary statistics with expression Quantitative Trait Loci (eQTLs) data. The most recent collected study that falls into this category is by van Rheenen et al. [41], where the authors conducted an enrichment analysis using a large European ancestry summary statistics dataset and disease-relevant cell types and tissue gene expression patterns from the Genotype–Tissue Expression (GTEx), as well as a co-expression based pathway analysis using Reactome, Gene Ontology and HPO terms. The authors reported, among others, membrane trafficking, intra-Golgi and retrograde Golgi-to-endoplasmic reticulum (ER) trafficking and macroautophagy as ALS-associated processes [41]. Iacoangeli et al. [44] conducted a *SCFD1*-centric gene functional enrichment approach, restricting their input set of genes only to those that were deemed to show significant levels of co-expression with *SCFD1*. We can observe a high level of reproducibility between the results of van Rheenen et al. [41] and Iacoangeli et al. [44], including the retrograde vesicle-mediated protein transport from the ER-to-Golgi, glutamatergic synapse and the regulation of synaptic vesicle docking and exocytosis. Another study that employed large meta-analysis summary statistics of European descent [20] and eQTLs was by Du et al. [46]. The authors report seven statistically significant KEGG gene sets related to peroxisome, TCA cycle, tight junction, PPAR signaling pathways, vesicular transport, arachidonic acid, glycolysis and gluconeogenesis metabolism [46]. In this case, we note a partial overlap with the results of van Rheenen et al. [41] and Iacoangeli et al. [44], mostly focused on vesicle-mediated transport. Du et al. study results are predominantly focused on central metabolism [46].

We further note several similarities between the GSA results of the previously mentioned studies and Saez-Atienzar et al. [45] who followed a Polygenic Risk Score gene-set analysis approach on European ancestry ALS cohorts as the previous studies. The authors report several developmental pathways, membrane trafficking, and signal transduction mediated by ribonucleotides as statistically significant gene sets [45]. Saez-Atienzar et al. [45] report a membrane trafficking process, which was also found as a statistically significant gene set term in the van Rheenen et al. study [41], and it is linked with intra-Golgi and retrograde Golgi-to-endoplasmic reticulum (ER) trafficking, as well as vesicular transport, terms also found as statistically significant by Du et al. [46]. The reported developmental pathways include among others, cell development, neuron projection morphogenesis, and neuron development [45], which we observe in the GSA results of mostly earlier studies, like the study by Deng et al. [48] who report several ALS-associated pathways, the majority of which relate to the nervous system development category. In addition, within the neuron development and membrane trafficking categories, we observe statistically significant terms (FDR < 0.05) in the Shang et al. study [52] including axon guidance, hedgehog signaling pathway and Wnt signaling pathway. The authors also report the autophagosome cellular component as statistically significant, a finding that aligns with the van Rheenen et al. statistically significant macroautophagy term [41].

Another interesting observation derives from the overlap between the highly statistically significant results of the previous (mostly of European descent) ALS GSA studies and the Chinese Han GS-GSA study by Xie et al. [53]. Specifically, among the 10 reported significantly ALS-associated pathways (FDR < 0.05) [53], we observe similar trends with the previous ALS studies including: neurodevelopmental pathways such as axon guidance, Wnt signaling pathway and neurotrophin signaling pathway, which controls synaptic function and plasticity, and is also associated with neuronal survival, morphology and differentiation [71]; lipid metabolism and membrane trafficking pathways such as the phosphatidylinositol signaling system, and the arachidonic acid metabolism [72]; immune system-related signaling pathways like the T-cell receptor signaling pathway; as well as key signaling pathways like the MAPK signaling pathway which is implicated in numerous cellular processes such as proliferation, differentiation and apoptosis.

Lastly, Benyamin et al. [42] assembled a cross-ancestry meta-analysis dataset which was then subjected to gene-level and gene-set level analysis. However, the study did not identify any statistically significant biological gene set associated with ALS, and only minimal information is provided regarding the GSA approach [42].

In summary, several biological pathways were reproduced among the collected ALS gene-set analyses, exhibiting particular interest in ALS pathology. Biological pathways related to “membrane trafficking”, “intra-Golgi and retrograde Golgi-to-endoplasmic reticulum (ER) trafficking”, “phosphatidylinositol signaling system”, “regulation of synaptic vesicle docking”, “exocytosis”, “autophagosome cellular component” and “macroautophagy” showed a higher reproducibility among the collected studies and play a key role in the pathology of ALS. We also note the presence of ALS-associated gene sets that concern nervous system development pathways as well as terms that were related to neuronal survival, morphology and differentiation, like cell development, neuron projection morphogenesis, neuron development and Hedgehog and Wnt signaling pathways.

## 6. Conclusions

In this review, we collected nine ALS gene-set analysis studies that employ GWAS datasets in order to understand the pathology of ALS in terms of functional pathways. We compared these studies in terms of their input datasets (type of data and cohort size), gene-set analysis approaches that they employed, possible multiple testing corrections and the main reported biological results.

We note that several studies provided only minimal information on their GSA approach, and others provided only the name of the software that was used. This lack of reported methodology contributes to low reproducibility, consistency and transparency across the ALS GS-GWAS studies.

Our current knowledge of ALS aetiology remains elusive. Genome-wide gene-set analysis has the potential to help us understand the complexity of this devastating disease, and how ALS pathology is interpreted in terms of molecular pathways. Several ALS studies approached these research questions through gene-set analysis. However, further advances are needed in order to fully uncover the underlying mechanisms of ALS for successful personalized disease and drug-targeting prediction approaches. From this survey, we identified several aspects that may be beneficial to bring together in subsequent work:

- The use of large cohort sizes can increase the power of genome-wide gene-set analyses;
- Comprehensive, transparent and reproducible genomic quality control strategies are likely to support more consistent biological findings;
- Data-driven and holistic approaches in the selection of genes and gene-set annotation databases are preferable;
- Selection of competitive GSA methods and mean-based statistics provide a better performance, and the biological assumptions are more consistent with a real-life complex functional network;
- Detailed and transparent GSA methodology can contribute to reproducible research results and informed decision-making;
- Enhanced visualisation approaches may aid interpretation, e.g., Enrichment Networks.

## Figures and Tables

**Table 1:** Current ALS GWAS studies that employ gene-set analysis (GSA) approaches. The ALS GWAS-GSA studies are described by the cohort size of input data, the ancestry of the genomic cohorts, the GSA software that is used, and, lastly, the source of the collected gene set annotations. The studies are sorted by chronological order of publication. ALS: Amyotrophic Lateral Sclerosis, BP: Biological Processes, eQTL: expression Quantitative Trait Loci, GO: Gene Ontology, GS: Gene Set, GSA: Gene-Set Analysis, GWAS: Genome-Wide Association Study, KEGG: Kyoto Encyclopedia of Genes and Genomes, MF: Molecular Function, MSigDB: Molecular Signatures Database, NA: Not Available, PRS: Polygenic Risk Score.

Studies	Input Data	Ancestry	GSA Software	GS Annotation
[41]	27,205 cases and 110,881 controls [41,42,43] eQTL data	European, Japanese, Chinese	FUMA, MAGMA, Downstreamer	G0, HPO, REACTOME
[44]	20,806 cases and 59,804 controls [14] eQTL data dbGaP Ac. phs000424.v8.p2	European	g:Profiler, Enrichr, GSEA	G0, KEGG
[45]	12,577 cases and 23,475 controls [20] 5605 cases and 24,110 controls [14] 2411 cases and 10,322 controls [14]	European	PRS approach	MSigDB
[46]	12,577 cases and 23,475 controls [20], eQTL data [47]	European	GSEA	KEGG
[42]	12,577 cases and 23,475 controls [20], 1234 cases and 2850 controls, 431 cases and 567 controls, [42]	European, Chinese, Australian	MAGMA	NA
[48]	276 ALS cases and 271 controls [49], 221 cases and 216 controls [50]	American, Irish	WebGestalt	GO
[51]	276 cases and 271 controls [49]	American	ICSNPathway	KEGG, BioCarta, GO BP, GO MF
[52]	276 cases and 271 controls [49], 221 cases and 211 controls [50]	American, Irish	WebGestalt	KEGG
[53]	250 cases and 250 controls	Chinese Han	WebGestalt	KEGG

**Table 2:** Current gene-set analysis (GSA) software employed by the collected ALS GWAS-GSA studies. Each GSA software is characterised by the type of input data, the null hypothesis (self-contained/competitive), and the gene-set method, employed by each study. eQTL: expression Quantitative Trait Loci, GS: gene set, GSA: Gene-Set Analysis, GWAS: Genome-Wide Association Studies, KS: Kolmogorov–Smirnov.

Software	Input Data	Null Hypothesis	GS Method	Studies
Downstreamer	*p*-values, eQTL	Competitive	Generalized least-squares regression	[41]
Enrichr	Gene list	Competitive	Overrepresentation/hypergeometric test	[44]
FUMA	eQTL & GWAS	Competitive	Overrepresentation	[41]
g:Profiler	Gene list	Competitive	Overrepresentation/hypergeometric test	[44]
GSEA/i-GSEA	*p*-values	Competitive	rank-based, (KS test)	[44,46,51]
MAGMA	Genotypes, *p*-values	Competitive	Linear regression	[42]
WebGestalt	Gene list	Competitive	Overrepresentation/hypergeometric test	[48,52,53]

**Table 3:** Main biological pathways that were discovered by current ALS GWAS-GSA studies. Here, we present the main significant findings of each study, in terms of ALS-associated gene sets with their accompanied *p*-values (where possible). We also include the chosen multiple testing correction methods and threshold for each study (where available), applied to the final list of significant findings. We note that, for practical purposes, this table does not include the full list of significant results for every study. The studies are sorted by chronological order of publication. ALS: Amyotrophic Lateral Sclerosis, ER: Endoplasmic Reticulum, GSA: Gene-Set Analysis, GWAS: Genome-Wide Association Study, NA: Not Available.

Studies	Multiple Testing Correction Method	Main Findings
[41]	Bonferroni	Cerebral cortical atrophy (*p*-value = 1.8 × 10^−8^), Abnormal nervous system electrophysiology (*p*-value = 4.1 × 10^−7^) Distal amyotrophy (*p*-value = 8.6 × 10^−7^), Membrane trafficking (*p*-value = 4.2 × 10^−6^), Intra-Golgi and retrograde Golgi-to-ER trafficking (*p*-value =1.4 × 10^−5^) Macroautophagy (*p*-value = 3.2 × 10^−5^)
[44]	FDR < 0.05	Vesicle-mediated transport in synapse (adjusted *p*-value = 7.58 × 10^−7^), Glutamatergic synapse (adjusted *p*-value = 4.20 × 10^−6^) Vesicle docking involved in exocytosis (adjusted *p*-value = 3.30 × 10^−5^)
[45]	FDR < 0.05	Neuron projection morphogenesis, Membrane trafficking, Signal transduction mediated by ribonucleotides
[46]	Empirical *p*-values	Peroxisome (empirical *p*-value = 0.006), Citrate cycle TCA cycle (empirical *p*-value = 0.025), Tight Junction (*p*-value NA) PPAR signaling pathway (empirical *p*-value = 0.025), SNARE interactions in vesicular transport (empirical *p*-value = 0.027), Arachidonic acid metabolism (empirical *p*-value = 0.040), Glycolysis-gluconeogenesis (empirical *p*-value = 0.043)
[42]	NA	No significant pathways were detected after multiple testing correction
[48]	NA	Nervous system development (adjusted *p*-value = 1.13 × 10^−9^)
[51]	FDR < 0.05	Chromatin assembly (FDR = 0.001), Nucleosome assembly (FDR = 0.018)
[52]	FDR < 0.05	RNA transport (adjusted *p*-value = 1.00 × 10^−3^), Vascular smooth muscle contraction (adjusted *p*-value = 1.80 × 10^−3^), Neuroactive ligand-receptor interaction (adjusted *p*-value = 6.30 × 10^−3^), Systemic lupus erythematosus (adjusted *p*-value = 6.30 × 10^−3^), Chemokine signaling pathway (adjusted *p*-value = 6.30 × 10^−3^), Hematopoietic cell lineage (adjusted *p*-value = 6.30 × 10^−3^), Cytosolic DNA-sensing pathway (adjusted *p*-value = 1.30 × 10^−2^), Protein processing in ER (adjusted *p*-value = 1.62 × 10^−2^), Alzheimer’s disease (adjusted *p*-value = 1.69 × 10^−2^), Parkinson’s disease (adjusted *p*-value = 3.12 × 10^−2^), Oxidative phosphorylation (adjusted *p*-value = 3.26 × 10^−2^), Cytokine–cytokine receptor interaction (adjusted *p*-value = 3.37 × 10^−2^)
[53]	FDR < 0.05	Phosphatidylinositol signaling system (adjusted *p*-value = 0.0011), Pathways in cancer (adjusted *p*-value = 0.0011), Wnt signaling pathway (adjusted *p*-value = 0.0020), Axon guidance (adjusted *p*-value = 0.0021), MAPK signaling pathway (adjusted *p*-value = 0.0021), Neurotrophin signaling pathway (adjusted *p*-value = 0.0021), Arrhythmogenic right ventricular cardiomyopathy (adjusted *p*-value = 0.0044), Colorectal cancer (adjusted *p*-value = 0.0099), Arachidonic acid metabolism (adjusted *p*-value = 0.0454), T-cell receptor signaling pathway (adjusted *p*-value = 0.0488)

## Data Availability

Not applicable.
